# Supplementary material for: A computational approach to map nucleosome positions and alternative chromatin states with base pair resolution
Source: eLife. 2016 Sep 13;5:e16970. doi: 10.7554/eLife.16970 (PMC5094857; doi:10.7554/eLife.16970)
Supplement: Supplementary file 2. — DOI: http://dx.doi.org/10.7554/eLife.16970.027 [file elife-16970-supp2.docx]

**Supplementary file 2A.** Cumulative distribution of the distance between *in silico* TBB positions and matched primary positions from *in silico* experiments. All distances are in the unit of base pairs.

|  |  | **Distance cumulative distribution** | | | | |
| --- | --- | --- | --- | --- | --- | --- |
| **Factor** | **Level** | **0.25** | **0.5** | **0.75** | **0.85** | **0.95** |
| Coverage | 82 | 1 | 2 | 3 | 4 | 5 |
| Coverage | 101 | 1 | 2 | 3 | 4 | 5 |
| Coverage | 113 | 1 | 1 | 3 | 4 | 5 |
| Coverage | 121 | 1 | 1 | 3 | 4 | 4 |
| Coverage | 129 | 1 | 1 | 3 | 5 | 5 |
| Coverage | 136 | 1 | 2 | 3 | 4 | 4 |
| Coverage | 144 | 1 | 2 | 3 | 3 | 4 |
| Coverage | 153 | 1 | 2 | 4 | 4 | 5 |
| Coverage | 164 | 1 | 1 | 4 | 5 | 5 |
| Coverage | 185 | 1 | 2 | 4 | 4 | 5 |
| Effective magnitude | 0.33 | 1 | 3 | 4 | 4 | 5 |
| Effective magnitude | 0.36 | 1 | 3 | 4 | 4 | 5 |
| Effective magnitude | 0.38 | 1 | 3 | 4 | 4 | 5 |
| Effective magnitude | 0.42 | 1 | 3 | 4 | 4 | 5 |
| Effective magnitude | 0.45 | 1 | 3 | 4 | 4 | 5 |
| Effective magnitude | 0.5 | 1 | 3 | 4 | 4 | 5 |
| Effective magnitude | 0.56 | 1 | 3 | 4 | 4 | 5 |
| Effective magnitude | 0.62 | 1 | 3 | 4 | 4 | 5 |
| Effective magnitude | 0.71 | 1 | 1 | 1 | 1 | 3 |
| Effective magnitude | 0.83 | 1 | 1 | 1 | 1 | 1 |
| Effective magnitude | 1 | 1 | 1 | 2 | 3 | 4 |
| Offset | 0 | 1 | 1 | 2 | 3 | 4 |
| Offset | 5 | 1 | 2 | 3 | 4 | 5 |
| Offset | 10 | 1 | 2 | 4 | 4 | 5 |
| Offset | 15 | 1 | 2 | 4 | 4 | 5 |
| Offset | 20 | 1 | 2 | 3 | 4 | 5 |
| Offset | 25 | 1 | 2 | 3 | 4 | 5 |
| Offset | 30 | 1 | 2 | 3 | 4 | 5 |
| Offset | 35 | 1 | 2 | 3 | 4 | 5 |
| Offset | 40 | 1 | 2 | 4 | 4 | 5 |
| Offset | 45 | 1 | 2 | 4 | 4 | 5 |
|  | | | | | | |

**Supplementary file 2B.** Cumulative distribution of the distance between *in silico* TBB positions and matched alternative positions from *in silico* experiments. All distances are in the unit of base pairs.

|  |  | **Distance cumulative distribution** | | | | |
| --- | --- | --- | --- | --- | --- | --- |
| **Factor** | **Level** | **0.25** | **0.5** | **0.75** | **0.85** | **0.95** |
| Coverage | 82 | 2 | 3 | 7 | 18 | 34 |
| Coverage | 101 | 2 | 3 | 6 | 14 | 33 |
| Coverage | 113 | 2 | 3 | 5 | 11 | 32 |
| Coverage | 121 | 1 | 4 | 5 | 9 | 29 |
| Coverage | 129 | 1 | 3 | 5 | 9 | 29 |
| Coverage | 136 | 2 | 3 | 4 | 8 | 28 |
| Coverage | 144 | 2 | 3 | 4 | 7 | 28 |
| Coverage | 153 | 1 | 3 | 5 | 7 | 28 |
| Coverage | 164 | 1 | 3 | 4 | 6 | 24 |
| Coverage | 185 | 1 | 3 | 5 | 5 | 22 |
| Effective magnitude | 0.33 | 1 | 3 | 4 | 4 | 5 |
| Effective magnitude | 0.36 | 1 | 3 | 4 | 4 | 5 |
| Effective magnitude | 0.38 | 1 | 3 | 4 | 4 | 5 |
| Effective magnitude | 0.42 | 1 | 3 | 4 | 5 | 6 |
| Effective magnitude | 0.45 | 1 | 3 | 4 | 5 | 7 |
| Effective magnitude | 0.5 | 1 | 3 | 4 | 5 | 9 |
| Effective magnitude | 0.56 | 1 | 3 | 4 | 6 | 15 |
| Effective magnitude | 0.62 | 2 | 3 | 6 | 11 | 28 |
| Effective magnitude | 0.71 | 1 | 4 | 18 | 28 | 38 |
| Effective magnitude | 0.83 | 8 | 19 | 33 | 38 | 43 |
| Offset | 5 | 2 | 3 | 4 | 5 | 7 |
| Offset | 10 | 2 | 4 | 7 | 8 | 10 |
| Offset | 15 | 2 | 3 | 8 | 13 | 14 |
| Offset | 20 | 1 | 3 | 5 | 17 | 19 |
| Offset | 25 | 1 | 3 | 5 | 22 | 24 |
| Offset | 30 | 1 | 3 | 5 | 26 | 29 |
| Offset | 35 | 1 | 3 | 4 | 29 | 33 |
| Offset | 40 | 1 | 3 | 4 | 6 | 38 |
| Offset | 45 | 1 | 3 | 4 | 6 | 43 |
|  | | | | | | |
