# Supplementary material for: A computational approach to map nucleosome positions and alternative chromatin states with base pair resolution
Source: eLife. 2016 Sep 13;5:e16970. doi: 10.7554/eLife.16970 (PMC5094857; doi:10.7554/eLife.16970)
Supplement: Supplementary file 3. — DOI: http://dx.doi.org/10.7554/eLife.16970.028 [file elife-16970-supp3.docx]

Supplementary file 3. A table comparison of published methods for determining nucleosome positions

| **Methods** | **Experimental approach** | **Computational approach** | **Overall performance** | **Limitations** | **Reference** |
| --- | --- | --- | --- | --- | --- |
| Parzen window | MNase-seq | • Smooth sequencing counts with window-average based approach  • Peak calling algorithm to identify nucleosome positions | • Estimate average nucleosome centers of relative broad nucleosome-containing regions  •50,000 – 70,000 nucleosome centers genome wide | • Sensitive to the window size used in the smoothing algorithms  • Difficult to resolve overlapping nucleosomes | Albert et al., 2007;  Valouev et al., 2008  Tsankov et al, 2010  Tirosh et al, 2012 |
| Template-based filtering | MNase-seq | • Identify templates for typical distributions of forward and reverse nucleosome reads  • Fit sequencing data to the best-correlated templates to determine nucleosome positions | • Estimate average nucleosome centers of relative broad nucleosome-containing regions  • ~64,000 nucleosome positions  • Improved center accuracy and identifying nucleosomes with variable sizes | • Difficult to resolve overlapping nucleosomes | Weiner et al, 2010 |
| NOrMAL | MNase-seq | • Modified Gaussian mixture model | •Comparable with template-based filtering method with improved performance | • Not designed to resolve overlapping nucleosomes  • Requires user defined parameters in the model | Polishko et al, 2012 |
| Chemical cleavage | Chemical cleavage of nucleosomal DNA followed by high throughput sequencing | • Identify characteristic cleavage pattern genome-wide  • Fit cleavage data with the identified patterns and calculate nucleosome centre positioning score | • Identify nucleosome positions at single base-pair resolution  • ~350,000 nucleosome positions genome-wide  • Resolve overlapping nucleosomes  • Periodic di-nucleotide pattern from identified nucleosome positions | • Due to its requirement of genetic engineering histone H4, this method is limited to budding and fission yeast thus far. | Brogaard et al, 2012  Moyle-Heyrman et al, 2013 |
| TBB approach | MNase-seq | • Estimate MNase digestion error from fragment sizes  • Template-based Bayesian approach to determine nucleosome positions | • Identify nucleosome positions at base-pair resolution  • ~125,000 nucleosome positions genome-wide  • Resolve overlapping nucleosomes  • Periodic di-nucleotide pattern from nucleosome positions  • Identify nucleosome positions present significantly in bulk population | • Require high Computational power  • Optimized for paired-end data | Current work |
| NucleoATAC | ATAC-seq  MNase-seq | Apply two-dimensional V-plot to identify nucleosome stricture based on fragment sizes and center locations | • Identify 13,000 – 16,000 nucleosome positions in broad open chromatin regions  • Resolve overlapping nucleosomes  • Observe periodic di-nucleotide pattern from nucleosome positions  • Infer both regulatory regions and nucleosome positions from ATAC-seq data | • Limited to broad open chromatin and regions  • Require relative high sequencing coverage for nucleosome determination  • Sensitive to the digested fragment length | Schep et al, 2015 |
| DNase-seq | DNase-seq | • Transform DNase-seq read counts into nucleosome score with Bayes-factor-based approach  • Identify nucleosome positions based on smoothed nucleosome scores | • Recapitulate global nucleosome profile similar to data generated from MNase-seq | • Assisted with known nucleosome position information in model training | Zhong et al, 2016 |
